# Variables Associated with a Urinary MicroRNAs Excretion Profile Indicative of Renal Fibrosis in Fabry Disease Patients

**DOI:** 10.1155/2019/4027606

**Published:** 2019-06-24

**Authors:** Sebastián Jaurretche, Germán Perez, Norberto Antongiovanni, Fernando Perretta, Graciela Venera

**Affiliations:** ^1^Biophysics and Human Physiology, School of Medicine. Instituto Universitario Italiano de Rosario, Rosario, Santa Fe, Argentina; ^2^Los Manantiales, Neurosciences Center, Grupo Gamma Rosario, Rosario, Santa Fe, Argentina; ^3^Faculty of Biochemical and Pharmaceutical Sciences, Nacional University of Rosario, Rosario, Santa Fe, Argentina; ^4^Gammalab, Grupo Gamma Rosario, Rosario, Santa Fe, Argentina; ^5^Center for Infusion and Study of Lysosomal Diseases, Instituto de Nefrología de Pergamino, Pergamino, Buenos Aires, Argentina; ^6^Intensive Care Unit, Hospital Dr. Enrique Erill, Belén de Escobar, Buenos Aires, Argentina; ^7^Research Department, Instituto Universitario Italiano de Rosario, Rosario, Santa Fe, Argentina

## Abstract

**Introduction:**

In advanced Fabry nephropathy stages, enzyme replacement theraphy (ERT) efficacy decreases, due to its impossibility to reverse renal fibrosis. Therefore, the finding of early kidney fibrosis biomarkers in affected patients is of interest. During renal fibrosis miR-21, miR-192 and miR-433 (fibrosis promotors) are activated by transforming growth factor-*β* (TGF-*β*), and miR-29 and miR-200 family (fibrosis supressors) are inhibited by TGF-*β*. The aim of this study is to analyze the probability that Fabry disease (FD) patients with some clinical variables can present an urinary microRNAs excretion profile indicative of renal fibrosis through a logistic regression analysis.

**Results:**

A population of 34 participants was included: 24 FD patients and 10 controls. 16/24 (66.66%) FD patients presented microRNAs urinary excretion profile indicative of renal fibrosis. This profile was observed by decrease of fibrosis suppresors miR-29 and miR-200 and not by increase of fibrosis promotors miR-21, miR192, and miR-433. Hypohidrosis, angiokeratomas, neuropathic pain, hearing loss, cardiac involvement, male gender, reduced *α*GalA activity, and renin-angiotensin-aldosterone system inhibitors treatment are associated with the appearance of amicroRNAs urinary excretion profile indicative of renal fibrosis. A probable beneficial effect on urinary microRNAs excretion profile was observed in patients receiving ERT with agalsidase beta. The correlation between parameters of renal function with each family of microRNAs was studied. The only association with statistical significance was found between miR-21 and urine albumin-creatinine ratio (p =0.021).

**Conclusions:**

A probable microRNAs regulation not mediated by TGF-*β* should be considered or TGF-*β* has a different effect in FD than in other nephropathies on microRNAs regulation. Typical clinical manifestations of classic FD are associated with appearance of urinary microRNAs profile indicative of renal fibrosis. FD patients express renal fibrosis biomarkers in urine prior to onset of pathological albuminuria. A direct correlation between urinary miR-21 and degree of albuminuria was observed.

## 1. Introduction

Fabry disease (FD, OMIM #301500) is a rare X-linked hereditary lysosomal storage disorder, caused by mutations in the GLA gene, encoding the acid hydrolase *α*-galactosidase-A (*α*GalA) enzyme, which catalyses neutral glycosphingolipids [1]. FD manifestations are consequence of glycosphingolipids accumulation in lysosomal, extralysosomal, and extracellular spaces [1, 2].

In renal tissue of affected patients, all cell types can be affected by abnormal globotriaosylceramide (Gb3) deposition from early stages of life [3]. Cellular dysfunction is believed to trigger a cascade of events including cellular death, compromised energy metabolism, small vessel injury, ion channel dysfunction, oxidative stress, impaired autophagosome maturation, toxic effect of Globotriaosylsphingosine (Lyso-Gb3), tissue ischemia, and, importantly, development of irreversible tissue fibrosis [1, 3]. Microalbuminuria is the first clinical manifestation of kidney damage that can be observed in affected patients [1]. However, irreversible renal histological lesions, including glomerulosclerosis and tubulointerstitial fibrosis, have been described in asymptomatic patients with normal estimated glomerular filtration rate (eGFR) and mild or absent proteinuria [3–6].

In Fabry nephropathy, the specific treatment effectiveness (enzyme replacement therapy: ERT) is greater if it is indicated in early stages, when irreversible histological lesions are not present. In these stages, cellular cleavage of Gb3 abnormally deposited has been reported, even in podocytes, although in this cell, the effect is dependent on accumulated dose of ERT administered [5]. In advanced renal damage stages, ERT efficacy decreases, due to its impossibility of being able to correct irreversible histological lesions, such as renal fibrosis [7, 8]. Any potential ERT benefit to ameliorate or reverse Fabry nephropathy progression is expected to take many years, especially if fibrosis is well established before ERT is started [7]. Therefore, the finding of early kidney fibrosis biomarkers in affected patients is of interest.

MicroRNAs (miRs; miRNAs) are a family of short noncoding RNAs that play important roles in posttranscriptional gene regulation. In the kidney, miRNAs play a role in the organogenesis and in the pathogenesis of several diseases, including renal fibrosis. During renal fibrosis Transforming growth factor-*β* (TGF-*β*) regulates expression of several microRNAs, such as miR-21, miR-29, miR-192, miR-200 and miR-433. MiR-21, miR-192, and miR-433 which are positively induced by TGF-*β* signaling play a pathological role in kidney diseases (fibrosis promotors). In contrast, both miR-29 and miR-200 families which are inhibited by TGF-*β* signaling protect kidneys from renal fibrosis (fibrosis supressors) [9].

Recently, we have reported that a profile of urinary microRNAs indicative of renal fibrosis is associated with decreased *α*Gal-A activity in young FD patients with mild or absent albuminuria [10]. In this FD population, the microRNAs profile indicative of renal fibrosis was associated with miR-29 and miR-200 decreased urinary excretion [10]. In another pilot study, it was observed that FD patients show decreased urinary excretion of both miR-29 and miR-200 compared with healthy controls [11]. Both studies conclude that a microRNAs urinary excretion indicative of renal fibrosis could be present in pre-albumin stages of Fabry nephropathy.

The aim of this study is to analyze the probability that FD patients with some clinical variables can present an urinary microRNAs excretion profile indicative of renal fibrosis through a logistic regression analysis. 

## 2. Material and Methods

### 2.1. Statement on Ethics

The study was carried out in accordance with the Declaration of Helsinki for Human Research and approved by the local Ethics Committee. Written informed consent for inclusion was obtained from each participant. Adult patients who met inclusion criteria signed informed consent. Pediatric patients agreed to participate and then their legal representative or guardian signed the informed consent.

### 2.2. Participants Characteristics

Patients with FD diagnosed by genetic test of any age and sex were included. Exclusion criteria: (i) patients with nephropathy by different aetiology than FD, (ii) patients who at the time of evaluation had symptoms unrelated to FD, which could alter the urinary excretion of microRNAs, and (iii) FD patients with inclusión criteria who refused to participate in the study. Elimination criteria: patients with inclusion criteria that presented some complication related to the collection process of the samples. A population of healthy subjects with similar demographic characteristics was included.

Blood samples and first morning urine were collected from the fasting participants.

All patients had a mutational study by direct sequencing and Multiplex Ligations Probe Amplification [12, 13], and quantification of *α*GalA enzymatic activity by fluorometric method [14]. Decreased or normal enzyme activity was considered at values < than or > than 4.0 nmol/h/l, respectively. Plasma and urine creatinine were determined by electrochemiluminescence (Roche Diagnostics). Albuminuria was determined by colorimetric method (Roche Diagnostics). The urinary albumin/creatinine ratio (uACR) was calculated to estimate 24 hour albuminuria [15]. Ratio values 0 to 30 were considered normal, 30 to 300 pathological albuminuria and > than 300 proteinuria in at least two samples. eGFR was calculated using CKD-EPI equation in adults [16] and Schwartz equation (modified in 2009) in pediatric patients [17]. To classify kidney disease, the recommendation of “Kidney Disease: Improving Global Outcomes Chronic Kidney Disease Guideline 2013 (KDIGO)” was used [18].

Peripheral nervous system (PNS) symptoms were considered by the presence of typical neuropathic pain crises and/or typical acroparesthesias and/or the demonstration of small neurological fibers damage by Quantitative Sensory Testing (QST) [1, 19]. Hypohidrosis and typical gastrintestinal (GI) symptoms were evaluated by questioning and physical examination [1, 19]. Dermatologist specialist in FD evaluated the presence of angiokeratomas [1, 19]. Hearing loss was defined by alterations of logoaudiometry test [1, 19]. Presence of cornea verticillata was evaluated by ophthalmological examination with slit lamp [1, 19]. Cardiac involvement was as follows: (1) cardiac fibrosis: presence of typical images in cardiac magnetic resonance imaging (MRI) with gadolinium and/or (2) cardiac ischemia: presence of typical changes in electrocardiogram and/or cardiac perfusion tests and/or (3) cardiac arrhythmia: presence of electrophysiological disorders in electrocardiogram; (4) left ventricular hypertrophy (LVI) assessed by tissue Doppler echocardiogram and/or cardiac MRI [1, 19]. Renal involvement was as follows: decreased eGFR and/or pathological albuminuria and/or proteinuria. Central nervous system (CNS) involvement: cerebral white matter lesions in cerebral MRI angiography and/or clinical stroke were considered by antecedents during the interrogation and physical examination and/or demonstration of lesion in cerebral MRI angiography [1, 19]. All patients studied on treatment with ERT were receiving agalsidase-beta (1mg / Kg /EOW).

In FD patients, microRNAs urinary excretion profile indicative of renal fibrosis was considered by increase of fibrosis activators miR-21, miR-192, or miR433 and/or decrease of fibrosis supressors miR29 or miR-200 compared to healthy subjects.

### 2.3. Urine Sample Preparation and MicroRNAs Extraction

Urine specimen was collected and sent to laboratory for processing immediately. A volume of 10 ml of urine sample was centrifuged at 3000 x g for 15 minutes. Nine ml of supernatant was discarded and the remaining milliliter was centrifuged at 15000 x g during 5 minutes. The urinary cell pellet was stored at -80°C until use.

microRNA molecules behave physicochemically different from the larger RNA molecules, and their isolation from biological samples requires validated procedures. The extraction of miRNAs was performed according to the manufacturer's protocol (NucleoSpin miRNA Plasma kits, Macherey-Nagel, Germany). This kit allows the simultaneous isolation of small RNA, large RNA and proteins in three separate fractions.

Currently, there is no available method that can assess the exact quantity or quality of small RNA and standard spectrophotometric methods to measure microRNA performance and quality are not suitable for biological samples. If the yield and concentration of miRNAs are sufficient, the evaluation of the quality of the extraction method can be performed by capillary electrophoresis or reverse transcription (RT) plus real-time polymerase chain reaction (qPCR) [20]. We evaluated the extractions by quantifying the small nucleolar RNA U6 by RT-qPCR. The reaction conditions are described below.

### 2.4. MicroRNAs RT-qPCR

To detect the urinary expression of miR-21, miR-29, miR-192, miR-200 families, and miR-433, reverse transcription (RT) reaction with a stem-loop primer was used [21]. Stem-loop RT primers were designed according to Chen et al. [22]. Sequence data was presented previously by us [10, 11].

The specificity of the stem-loop RT primers of each miRNA is given by an extension of six nucleotides at the 3′ end; this extension is inverse and complementary to the last six nucleotides of the 3′ end of the miRNA.

microRNAs were reverse transcribed using Transcriptor First Strand cDNA Synthesis Kit (Roche Diagnostics). Briefly, 5 *μ*l total eluate was mixed with 1 *μ*M stem-loop RT primer, 0,5 *μ*M dNTPs, 1x RT reaction buffer, 20 U RNase inhibitor, and 10 U Transcriptor RT and made up to 20 *μ*l with H2O. RT was performed at 16°C for 30 minutes, 42°C for 30 minutes, 60°C for 60 minutes, and 70°C for 15 minutes. The resulting cDNA was stored at -80°C until use.

FastStart Universal SYBR Green Master/ROX (Roche Diagnostics) was used for the qPCR reaction, which was performed according to the manufacturer's protocol on a StepOne Plus System (Applied Biosystems). cDNA was amplified using a miRNA-specific forward primer and the universal reverse primer [10]. The forward primers are sequence specific for each miRNA but do not contain the last six nucleotides of the 3′ end of the miRNA. To improve the melting temperature, 5 to 7 nucleotides were added at the 5′ end [10].

RT-qPCR was carried out in compliance with the MIQE guidelines [23]. All qPCR reactions were performed in duplicate, followed by melt curve analysis to verify their specificity and identity. Small nucleolar RNA U6 was selected as the endogenous reference control [24]. Relative microRNA expression levels were calulated using the 2-ΔΔCt method as previously described [25].

### 2.5. Data Analysis

Normal distribution of continuous variables was tested using the Shapiro-Wilk Test. For continuous variables, comparison of means/medians was performed using Student-t test for variables that followed a normal distribution and Mann-Whitney test/related samples Wilcoxon Signed Rank test for variables who did not. If the qualitative variable had more than two categories, an ANNOVA test was used for variables with normal distribution, and Kruskal-Wallis test was used for those without. For categorical variables, the comparison of the variables distribution between groups was done using the Chi-square or Fisher exact tests. Confidence interval was of 95%. Values of p <0.05 were considered of statistical significance. Each statistical test was applied for each family of microRNA studied.

The probability that FD patients present a profile of urinary microRNAs excretion indicative of renal fibrosis was estimated with a binary logistic regression model.

The presence of this profile indicative of renal fibrosis as a dichotomous variable according to the degree of albuminuria was analyzed to evaluate its usefulness as an early biomarker of kidney damage in nonalbuminuric patients.

## 3. Results

A population of 34 participants was included; 24 FD patients and 10 controls.  Table 1 shows the demographic characteristics of controls versus FD patients.

Twenty-three of 24 FD patients (95.83%) presented symptoms of classic FD phenotype. Twelve pediatrics FD patients (7 girls/5 boys; age: 10.33±3.93 years; eGFR: 152.33±48.39 ml/min/1.73 m^2^; uACR: 21.75 ± 37.13 mg/g) and 12 adult FD patients (9 females/3 males; age: 37.08±14.74 years; eGFR: 109.90±29.34 ml/min/1.73 m2; uACR: 32.93±30.86 mg/g) were included.

**Figure 1:**
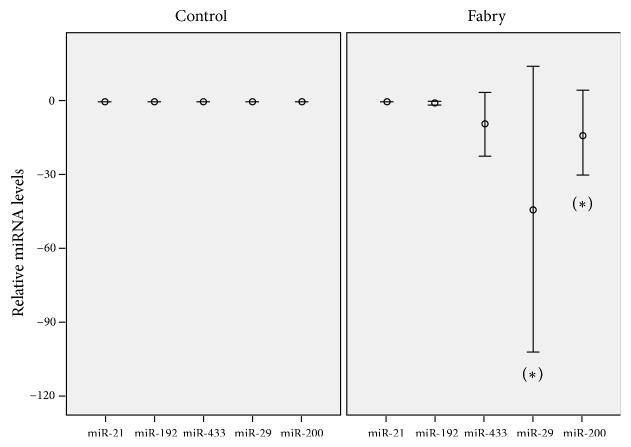
*Relative expression levels of miRNAs in urinary sediment of controls and FD patients*. Bars represent 2^−∆∆Ct^ values calculated by Delta-Delta Ct (∆∆Ct) method (Confidence interval: 95%). Expression was normalized to U6, and data are represented as means ± SEM. (*∗*) p = < 0.005.

Figure 1 shows the relative expression levels of miRNAs in urinary sediment of controls and FD patients. 16/24 (66.66%) FD patients presented microRNAs urinary excretion profile indicative of renal fibrosis. This profile was observed by decreasing of both fibrosis suppresors (miR-29 and miR-200) and not by increase of fibrosis promotors (miR-21, miR192 and miR-433) (Figure 1).

**Figure 2:**
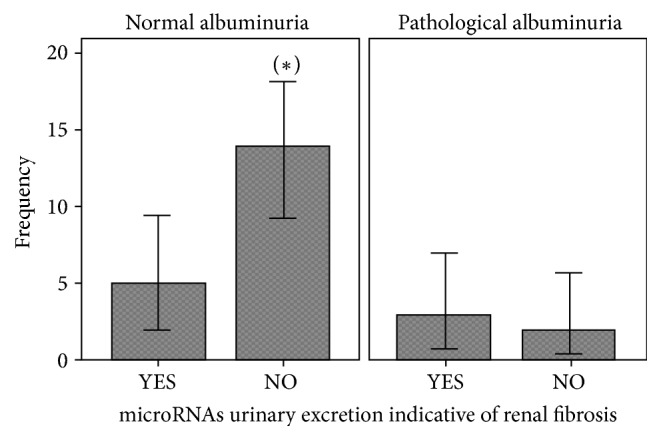
*Urinary microRNAs excretion profile indicative of renal fibrosis according to degree of albuminuria in Fabry disease patients*. Error bars indicates 95% confidence interval. (*∗*) p = < 0.005.

A highest frequency of urinary microRNAs indicative of renal fibrosis was observed in FD patients with normal albuminuria and a lower frequency was found in FD patients with pathological albuminuria (Figure 2).

Table 2 shows the frequency of clinical manifestations and its association with urinary microRNAs indicative of renal fibrosis in FD patients.

Binary logistic regression model was able to predict the appearance of urinary microRNAs indicative of renal fibrosis in FD patients.  Table 3 shows a model summary.

**Figure 3:**
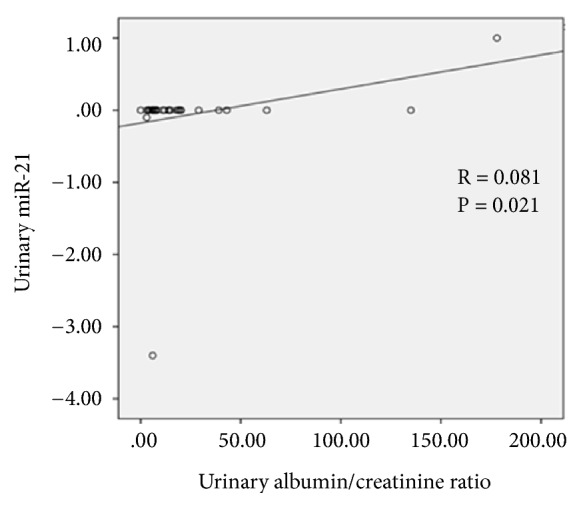
Linear correlation between urinary miR-21 and urinary albumin/creatinine ratio in Fabry disease patients.

The correlation between uACR and eGFR with each family of microRNAs was studied. The only one association with statistical significance was found between miR-21 and uACR (p =0.021) (Figure 3). There was no statistically significant correlation between uACR and eGFR with the rest of miR studied (uACR/miR-192, p = 0.790; uACR/miR-433, p = 0.933; uACR/miR-29, p = 0.536; uACR/miR-200, p = 0.766; eFGR/miR-21, p = 0.093; eGFR/miR-192, p = 0.047; eGFR/miR-433, p = 0.122; eGFR/miR-29, p = 0.408; eGFR/miR-200, p = 0.385).

## 4. Discussion

Renal fibrosis is a feature of Fabry nephropathy and its early development has been reported in renal biopsies of affected patients, even with normal eGFR and without pathological albuminuria [3–7]. The time-course of kidney fibrosis is not clearly established, but emerging evidence points are early podocyte injury and fibrosis generated by epithelial cells that increase as disease progresses [3, 5, 7, 26, 27].

The present work is an analysis of FD patients with mild nephropathy (preserved eGFR and mild or absent albuminuria) compared with healthy subjects of similar characteristics.

Our results show that miR-29 and miR-200 are decreased in urine of FD patients compared with healthy subjects while the miR-21, miR-192 and miR-433 are similarly expressed. These results, in a larger population of pediatric and adult FD patients, replicate our previous findings when only pediatric affected patients were included [10]. According to these results, a probable microRNAs regulaction not mediated by TGF-*β* should be considered, or that TGF-*β* has a different effect in FD than in other nephropathies on microRNAs regulation [10, 11].

Lyso-Gb3, a deacylated Gb3, is increased in plasma of FD patients [1]. The deleterious Lyso-Gb3 effects on podocytes and renal tubular cells have been demonstrated in both animal and human models of Fabry nephropathy [28, 29]. In podocytes, exposure to Lyso-Gb is correlated with increased expression of TGF-*β* and extracellular matrix components, both mechanisms associated with renal fibrosis development [28]. In renal tubular cells exposed to Lyso-Gb3, epithelial-mesenchymal transition has been described as another mechanism related to renal fibrosis [29]. The role of Lyso-Gb3 or other harmful molecules and growth factors different than TGF-*β* should be studied to explain this urinary microRNAs expression that appear to be different from a regulation TGF-*β* mediated.

In a previous pilot study on the subject, we found that miR-21, miR-29, miR-192, miR-200, and miR-433 urinary excretion was similar between healthy subjects and two FD patients subgroups: (i) women with mild FD phenotype and normal *α*GalA activity and (ii) women with *α*GalA normal and more severe phenotype who were receiving ERT [11]. In the same study, we found a urinary decrease of miR-29 and miR-200 in males of any age, with normal eGFR and without pathological albuminuria [11]. In the present work a statistical model was designed to predict the probability that FD patients with certain clinical variables may be developing a urinary microRNAs excretion profile indicative of renal fibrosis.

In our population, we found through the regression analysis that hypohidrosis, reduced *α*Gal-A activity, angiokeratomas, male gender, neuropathic pain, hearing loss, cardiac involvement, and renin-angiotensin-aldosterone system (RAAS) inhibitors treatment are clinical variables associated with urinary microRNAs excretion profile indicative of renal fibrosis. Except RAAS inhibitors treatment, all variables capable of predicting the appearance of urinary microRNAs indicative of renal fibrosis are typical manifestations of classic FD [19]. Classic FD (or type 1) is the most severe disease phenotype that is caused by serious deficiency of *α*GalA activity and tissue deposition of nonmetabolized substrates from fetal stages, with clinical manifestations since childhood and appearance of renal, cardiac, and cerebrovascular disease in affected young adult patients, all this associated with greater morbidity and mortality and shortening of life expectancy in affected patients of both genders [30].

Regarding the variables related to FD treatment, it is an expected result that ERT does not correlate with a microRNAs profile related to renal fibrosis. That is to say, a probable beneficial effect was observed in patients who receiving ERT. A paradoxical result is the correlation between RAAS inhibitors treatment and a urinary microRNAs indicative of renal fibrosis. Probably this association could be explained because the treatment with this drugs is initiated by nephrologists in presence of nephropathy (pathological albuminuria or reduced eGFR), when tissue damage is already advanced.

In our study, a significant proportion of nonalbuminuric patients had urinary microRNAs indicative of renal fibrosis. This finding could mean that FD patients express renal fibrosis biomarkers in urine prior to onset of pathological albuminuria. This could mean that urinary miRs are excreted in a similar way in FD and in other forms of chronic kidey disease (CKD), in which microRNAs urinary excretion decreases with the kidney disease progression [30].

Finally, when individual microRNAs families with other renal function parameters were analyzed, the only association found was the direct correlation between urinary miR-21 and albuminuria, although with a linear correlation of scarce significance. This is an expected finding, since miR-21 is a well-known molecule that promotes fibrosis in several CKD models of any cause. This finding should be analyzed in a greater number of patients, including a higher proportion of patients with pathological albuminuria.

Although the small size of the population studied and the cross-sectional design may represent a statistical limitation, other studies devoted to similar purposes were carried out with a similar or smaller number of patients [4, 5, 31–33].

## 5. Conclusions

Fibrosis supressors miR-29 and miR-200 are decreased in urine of FD patients compared with healthy subjects, while the fibrosis activators miR-21, miR-192, and miR-433 are similarly expressed. According to these results, a probable microRNAs regulation not mediated by TGF-*β* should be considered or TGF-*β* has a different effect in FD than in other nephropathies on microRNAs regulation.

Typical clinical manifestations of classic FD as hipohidrosis, angiokeratomas, neuropathic pain, hearing loss, cardiac involvement, male gender, and reduced *α*GalA activity are associated with the appearance of a urinary microRNAs excretion profile indicative of renal fibrosis.

A probable beneficial effect on urinary microRNAs excretion profile was observed in patients who receiving ERT with agalsidase beta.

FD patients express renal fibrosis biomarkers in urine prior to onset of pathological albuminuria but the urinary microRNAs excretion decreases with the nephropathy progression. In this sense, the profile of urinary excretion of microRNAs indicative of renal fibrosis could be useful as an early biomarker of renal fibrosis in the prealbuminuric stage but they would not be useful as biomarkers of evolution, although this hypothesis should be confirmed in longitudinal studies.

A direct correlation between urinary miR-21 and degree of albuminuria was observed. It could be hypothesized that this miR individually could be useful as a biomarker of Fabry nephropathy progression, although again, this finding should be confirmed in longitudinal studies.

## Figures and Tables

**Table 1:** Demographic characteristics of controls versus Fabry disease patients.

	Controls	FD patients	*p* value
N	10	24	-
Gender	4M/6F	7M/17F	-
Age*∗*	27.00±14.77	23.70±17.26	0.602
uACR*∗*	9.10±5.78	24.67±34.26	0.166
eGFR*∗*	118.12±19.44	136.19±36.74	0.153
Genotype	-	E398X;R363H; L415P;R227Q; R301Q; del3&4exon; L106R;C238Y	-

(*∗*) median ± standard desviation

References: M: male; F: females; uACR: urinary albumin/creatinine ratio; eGFR: estimated glomerular filtation rate.

**Table 2:** Frequency of clinical manifestations and association with urinary microRNAs excretion profile indicative of renal fibrosis in Fabry disease patients.

	Frequency (%)	Association with microRNAs urinary excretion profile indicative of renal fibrosis. Pearson correlation (*p* value)
Reduced *α*GalA activity	9/24 (37.50)	0.548 (0.006)^*∗∗*^
Male gender	7/24 (29.16)	0.454 (0.026)^*∗*^
Neuropathic pain	13/24 (54.16)	0.414 (0.044)*∗*
Hipohidrosis	10/24 (41.66)	0.598 (0.002)^*∗∗*^
GI symptoms	11/24 (45.83)	0.296 (0.161)
Angiokeratomas	12/24 (50.00)	0.530 (0.008)^*∗∗*^
Hearing loss	6/24 (25.00)	0.408 (0.048)^*∗*^
Pathological Albuminuria	5/24 (20.83)	-0.29 (0.169)
Reduced eGFR	0/24 (0.00)	- (+)
Cardiac involvement	6/24 (25.00)	0.408 (0.048)*∗*
CNS involvement	5/24 (20.83)	0.363 (0.081)
ERT treatment	9/24 (37.50)	0.365 (0.079)
RAAS inhibitors Treatment	6/24 (25.00)	0.408 (0.048)^*∗*^

^*∗*^ The correlation is significant at the 0.05 level (bilateral)

^*∗∗*^ The correlation is significant at the 0.01 level (bilateral)

(+) Can not be calculated because the “reduced eGFR” variable is constant

References: *α*GalA: *α*-galactosidase-A; GI: gastrointestinal; eGFR: estimated glomerular filtration rate; CNS: nentral nervous system; ERT: enzyme replacement theraphy; RAAS: renin-angiotensin-aldosterone system.

**Table 3:** Binary logistic regression model to predict the appearance of urinary microRNAs excretion profile indicative of renal fibrosis in Fabry disease patients.

Explanatory variable (+)	Score in the model	p value
Hipohidrosis	8.571	0.003 (*∗*)
Reduced *α*GalA activity	7.200	0.007 (*∗*)
Angiokeratomas	6.750	0.009 (*∗*)
Male gender	4.941	0.026 (*∗*)
Neuropathic pain	4.112	0.043 (*∗*)
Hearing loss	4.000	0.046 (*∗*)
Cardiac involvement	4.000	0.046 (*∗*)
RAAS inhibitors treatment	4.000	0.046 (*∗*)
ERT	3.200	0.074
Genotype	3.553	0.059
CNS involvement	3.158	0.076
Age	2.416	0.120
GI symptoms	2.098	0.148
Pathological albuminuria	2.021	0.155

Chi squared = 0.002; Cox y Snell R^2^ = 0.720; Nagelkerke R^2^ = 1.000. Global percentage correctly classified = 100%.

(+) Explanatory variables with greater capacity to predict the appearance of microRNAs urinary extretion profile indicative of renal fibrosis, classified in decreasing order.

(*∗*) Explanatory variables able to predict the appearance of urinary microRNAs extretion profile indicative of renal fibrosis.

References: *α*GalA: *α*-galactosidase-A; RAAS: renin-angiotensin-aldosterone system; ERT: enzyme replacement theraphy; CNS: nentral nervous system; GI: gastrointestinal.

## Data Availability

The data used to support the findings of this study are included within the article.
